# Attitudes toward and beliefs about obese persons across Hong Kong and Taiwan: wording effects and measurement invariance

**DOI:** 10.1186/s12955-019-1198-6

**Published:** 2019-07-30

**Authors:** Meng-Che Tsai, Carol Strong, Janet D. Latner, Yi-Ching Lin, Amir H. Pakpour, Chung-Ying Lin, Shu-Mei Wang

**Affiliations:** 1 Department of Pediatrics, National Cheng Kung University Hospital, College of Medicine, National Cheng Kung University, Tainan, Taiwan; 2 Department of Public Health, National Cheng Kung University Hospital, College of Medicine, National Cheng Kung University, Tainan, Taiwan; 3 Department of Psychology, University of Hawaii at Manoa, Honolulu, Hawaii USA; 4 Department of Early Childhood and Family Education, College of Education, National Taipei University of Education, Taipei, Taiwan; 5 Social Determinants of Health Research Center, Qazvin University of Medical Sciences, Qazvin, Iran; 6 Department of Nursing, School of Health and Welfare, Jönköping University, Jönköping, Sweden; 7 Department of Rehabilitation Sciences, Faculty of Health and Social Sciences, The Hong Kong Polytechnic University, 11 Yuk Choi Rd, Hung Hom, Hong Kong

**Keywords:** Cross-culture, Psychometrics, Weight bias, Weight stigma, Young adults

## Abstract

**Background:**

The psychosocial consequences of obesity are important but often underrated. The Attitudes Toward Obese Persons (ATOP) and Beliefs About Obese Persons (BAOP) scales used to measure weight-related bias have little psychometric information, especially in East Asian contexts. The objective of this study was to use rigorous statistical methods to demonstrate the psychometric properties of these two instruments in Hong Kong and Taiwanese college students.

**Methods:**

A convenience sample of 707 students was recruited from the universities in Hong Kong and Taiwan. Several competing confirmatory factor analyses (CFAs) were conducted to confirm the factorial structure of the ATOP and BAOP. The best fit models for the ATOP and BAOP were chosen for the examination of the measurement invariance across subcultures. We then compared configurable models with or without loading and/or intercept constrained before correlating the latent constructs between the best models for the ATOP and BAOP.

**Results:**

The comparison in multiple CFAs found that the model with one factor and two correlated-wording-method factors outperformed the other models for both the ATOP and BOAP. However, the internal consistency was suboptimal (ATOP: α = .56 to .80; BTOP: α = .57 to .65) and the measurement invariance was somewhat unsupported among the Hong Kong and Taiwan samples. Moreover, after controlling wording effects, the latent construct of the ATOP was moderately associated with that of BAOP (*r* = .356; *p* < .001).

**Conclusion:**

Chinese versions of the ATOP and BAOP can be treated as a unidimensional factor for use in Hong Kong and Taiwan university students. However, further refinements of both instruments may be needed before using them to capture the social attitudes and beliefs toward obesity individuals, which is expected to advance our understanding of weight-related bias in East Asian contexts.

## Background

Obesity is a serious global epidemic [1], including in many East Asian countries. For example, 35% of Hong Kong adults are overweight [2] and 43% of Taiwanese adults are overweight [3]. These rising rates pose public health challenges given the negative effects of overweight and obesity on physical health (e.g., high risk of cardiovascular disease, type II diabetes, and hypertension) [4, 5] and psychosocial well-being (e.g., high risk of depressive symptoms and decreased quality of life) [6–8]. However, the psychosocial consequences of obesity are often underestimated. For example, weight-based teasing, bullying, and discrimination may, in turn, cause internalizing or externalizing emotional problems. Previous research has shown that being teased or bullied because of weight was associated with higher odds of depression or suicidal ideation in obese youth [9]. Moreover, empirical studies revealed that people with obesity had a lower chance than their normal-weight counterparts of being recruited by employers [10]. In addition, healthcare providers have been shown to have negative attitudes toward people with obesity [10], which may jeopardize their treatment quality. Given that weight bias (or weight stigma) is a major contributor to psychosocial difficulties affecting people with obesity, it is imperative that public health professionals begin to tackle this issue. As revealed in recent studies, Hong Kong and Taiwan populations have a similar issue on weight-related stigma, which is linked to negative psychological outcomes [11–13].

Given its critical role, studies measuring weight bias (including the biased attitude toward and beliefs about people with obesity) have been expanding in recent decades [14–16]. The Attitudes Toward Obese Persons (ATOP) and Beliefs About Obese Persons (BAOP) scales are commonly used instruments; however, only two studies have thoroughly examined their psychometric properties [17, 18]. Additionally, other studies have revised the ATOP and BAOP into different versions of factorial structures [19–21]. However, to the best of our knowledge, earlier works reported a three-factor structure for the ATOP and a one-factor structure for BAOP [17, 18], but no studies have conducted a confirmatory factor analysis (CFA) to examine the factor structures of the ATOP and BAOP. Specifically, as most studies treated the ATOP as a unidimensional tool [22, 23], we should obtain evidence showing that the ATOP can be used based on a one-factor structure. Furthermore, no studies have considered the wording effects of these instruments (i.e., the positively worded and negatively worded items used in the ATOP and BAOP) or measurement invariance issues. Given that several studies have identified the influences of wording effects on the factorial structure of an instrument [24–27], we should not ignore the potential wording effects in the ATOP and BAOP when examining their factorial structures. We then hypothesized that the factorial structures of the ATOP and BAOP would be more consolidated if potential wording effects were accounted for in the CFA models.

Another important psychometric issue is measurement invariance; that is, whether the scale assesses the same construct in two different populations, including factorial structures and item descriptions. A prerequisite to comparing or combining the instrument scores between two populations is that the instrument is measurement equivalent across the two populations [28, 29]. Measurement invariance is extremely important when conducting cross-cultural studies or subcultural analysis. Therefore, Hong Kong and Taiwan, which share the Chinese culture but have developed distinctive subcultures due to previous history of different colonization (Hong Kong used to be governed by the United Kingdom and Taiwan used to be governed by Japan), could be a valuable venue for developing and examining the psychometric properties of the ATOP and BAOP across these two areas. Importantly, if the ATOP and BAOP are measurement invariant across Hong Kong and Taiwan subcultures, future studies on weight bias can confidently use both instruments to describe and compare attitudes toward and beliefs about people with obesity.

The study purpose is to translate, adapt, and examine the psychometric properties of the Chinese versions of the ATOP and BAOP in adult samples recruited in Taiwan and Hong Kong. We applied rigorous statistical methods (i.e., competing CFA models) to demonstrate the properties of the two instruments. Moreover, in the psychometric testing, we carefully considered the issues of wording effects and measurement invariance across Hong Kong and Taiwan samples.

## Methods

The study was approved by the Ethics committee of The Hong Kong Polytechnic University (Ref number: HSEARS20161214002) and the procedures were carried out in accordance with the Declaration of Helsinki. All participants received clear information about the study, fully understood the study purpose, and all signed a written informed consent.

### Participants and study design

We used convenience sampling and cross-sectional design to recruit both Hong Kong and Taiwan participants between March and July 2017. Hong Kong participants were from one university (located in Kowloon), and the Taiwan participants were from five universities (one located in Northern Taiwan, one in Central Taiwan, and three in Southern Taiwan). The inclusion criteria were (1) aged over 18 years; (2) agreed to participate; (3) understood written Chinese in traditional characters. The exclusion criteria were (1) had cognitive impairments or had difficulties in understanding questionnaires; (2) had a physical disability that causes difficulties in answering questionnaires.

For the recruitment in Hong Kong, the corresponding author contacted two colleagues teaching in health-related program (both are in occupational therapy) and one colleague not in health-related program (in mechanical engineering) to assist in inviting participants. For the recruitment in Taiwan, the second author contacted one acquaintance in Central Taiwan and another in Southern Taiwan to invite participants from their *Introduction to Medical Management* and *Introduction to Epidemiology* courses. The second author also invited participants from her *Introduction to Psychology* course. The fourth author recruited participants from her *Developmental Psychology* and *Introduction of Family Life Education* courses in Northern Taiwan. After obtaining the approval from the university professors to distribute questionnaires during their class, several research assistants (or the professors) used the last 20 min of a class to describe the study purpose and recruit participation. If the students were willing to participate, they first signed a written informed consent, and then completed a background information sheet, the ATOP, and the BAOP. In total, 400 students in Hong Kong and 307 in Taiwan turned in the written informed consents and the questionnaires.

We adopted the rule of thumb in factor analysis (i.e., 15 cases per item) to determine our sample size. Given that the ATOP has 20 items and the BAOP has 8 items, we used the item number in the ATOP for sample size estimation: 20 multiplied by 15 equals to 300 participants. Because we were unsure whether Hong Kong and Taiwan participants could be analyzed together, we proposed to have 300 participants in each area. Thus, the sample size of 400 in Hong Kong and that of 307 in Taiwan were sufficient for our psychometric testing.

### Translation procedure for the ATOP and BAOP

After contacting the developer of the ATOP and BAOP (Prof. Allison), we learned that both instruments had never been translated into Chinese, and we obtained the permission to translate them. In order to ensure their linguistic validity, we adopted a standard translation procedure including forward translation, back translation, and reconciliation [30, 31]. Two independent Hong Kong translators who were fluent in English and were majoring in psychology did the forward translations. After receiving the two independent translations, the corresponding author worked with a research assistant with a Bachelor degree in psychology to reconcile the two forward translations. The back translation was done by one mainland Chinese translator with a bachelor’s degree in English who has been living in the U.S. in an immersion program for 1 year. The third author compared the back translation to the original version and provided additional comments to revise. After revising all the comments, the final translated versions of the ATOP and BAOP were circulated among the first, second, and corresponding authors to ensure its readability for Taiwan and Hong Kong populations.

Given the similarity in Hong Kong and Taiwanese cultures, we did not further adopt the approach of transadaptation to modify the questionnaires so that we could evaluate the cross-cultural psychometric properties of the translated ATOP and BAOP.

### Instruments

Demographics were assessed using a background information sheet that asks the age, gender, height, weight, major in the university, and self-perceived weight status (underweight, normal-weight, or overweight).

The Attitudes Toward Obese Persons Scale contains 20 items rated on a six-point Likert-type scale (− 3 = *strongly disagree* to 3 = *strongly agree*). The ATOP was originally adapted from the Attitudes Toward Disabled Persons Scale [32]. After reverse coding the 13 negatively worded items, summing the 20-item scores and adding 60 to the summated score, a higher score indicates more positive attitudes toward people with obesity [17]. Three factors (Different Personality; Social Difficulties; Self-Esteem) have been extracted from the ATOP scale in the original version [17] and the Turkish version [18]. However, the internal consistency has never been tested for each factor, and most studies treat the ATOP as a unidimensional scale [22, 23]. In terms of the entire ATOP, the internal consistency ranged between 0.80 and 0.84 [17].

The Beliefs About Obese Persons Scale, a scale measures the extent that an individual believes obesity is under the control of a person with obesity, contains 8 items rated on a six-point Likert-type scale (− 3 = *strongly disagree* to 3 = *strongly agree*). After reverse coding the 6 negatively worded items, summing the 8-item scores and adding 24 to the summated score, a higher score indicates stronger beliefs that people with obesity cannot control their weight status [17]. The BAOP is treated as a unidimensional scale [22, 23], and the one-factor structure has been supported in its original and Turkish versions [17, 18]. In addition, the internal consistency of the BAOP ranged between 0.65 and 0.82 [17].

### Data analysis

We first separately present the characteristics of Hong Kong and Taiwan participants using mean (for continuous data) or frequency (for categorical data). Then, we compared the differences in the characteristics between Hong Kong and Taiwan participants using independent t-test (for continuous data with normal distribution), Mann-Whitney test (for continuous data with non-normal distribution) or *χ*^*2*^ test (for categorical data). Using the Shapiro-Wilk test, age, body mass index (BMI), and BAOP score were found to be non-normally distributed in both Hong Kong and Taiwan samples; ATOP score was normal distributed in both Hong Kong and Taiwan samples. Moreover, we used Cohen’s *d* to present the effect size of the differences in ATOP and BAOP scores, where a value > 0.2 indicates nonnegligible [8]. Afterward, we calculated the internal consistency using Cronbach’s α and McDonald’s ω, where > 0.7 indicates satisfactory [18], for both the ATOP (including the three factors and the entire the ATOP) and the BAOP.

**Fig. 1:**
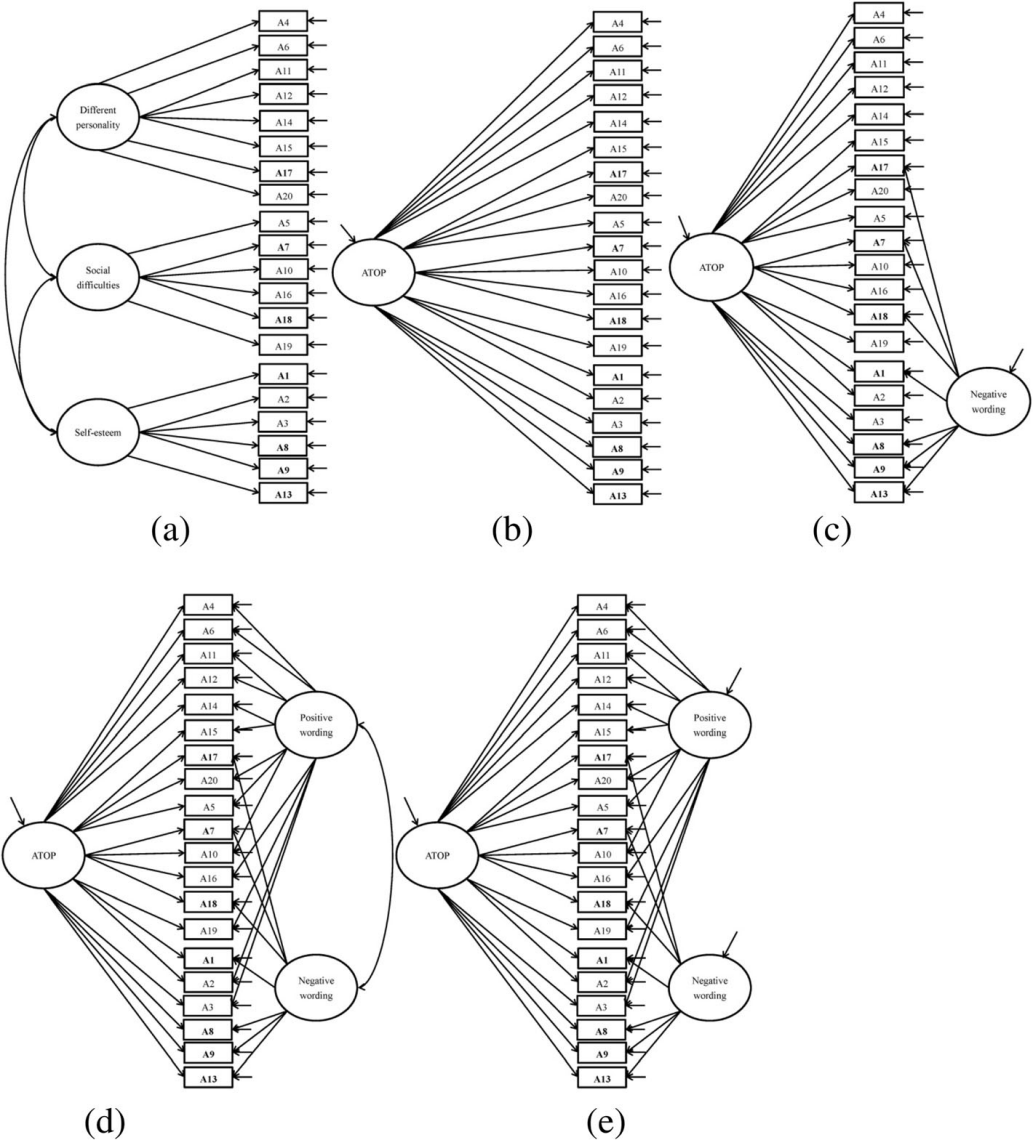
Structure of Attitude Toward Obese Persons Scale (ATOP). **a** Three-factor solution model (Different Personality, Social Difficulties, and Self-Esteem) without wording factors. **b** One-factor solution model (ATOP) without wording factors. **c** One-factor solution model (ATOP) with one method effect (negative wording). **d** One-factor solution model (ATOP) with two correlated method effects (negative wording and positive wording). **e** One-factor solution model (ATOP) with two uncorrelated method effects (negative wording and positive wording). Negatively worded items are in **bold**

Several CFAs with a diagonal weighted least squares (DWLS) estimator were conducted to confirm the factorial structure of the ATOP and BAOP. In the ATOP, we compared five models to understand its structure: a three-factor structure (Different Personality, Social Difficulties, and Self-Esteem) without wording factors (Fig. 1a), a one-factor structure (ATOP) without wording factors (Fig. 1b), a one-factor structure (ATOP) with one wording factor (negative wording; Fig. 1c), a one-factor structure (ATOP) with two correlated wording factors (positive and negative wordings; Fig. 1d), and a one-factor structure (ATOP) with two uncorrelated wording factors (positive and negative wordings; Fig. 1e). We did not test the three-factor structure with wording factor(s) because this would make the CFA model too complicated and might violate the principle of parsimony. In addition, most studies apply the ATOP as a one-factor structure rather than a three-factor structure [22, 23].

**Fig. 2:**
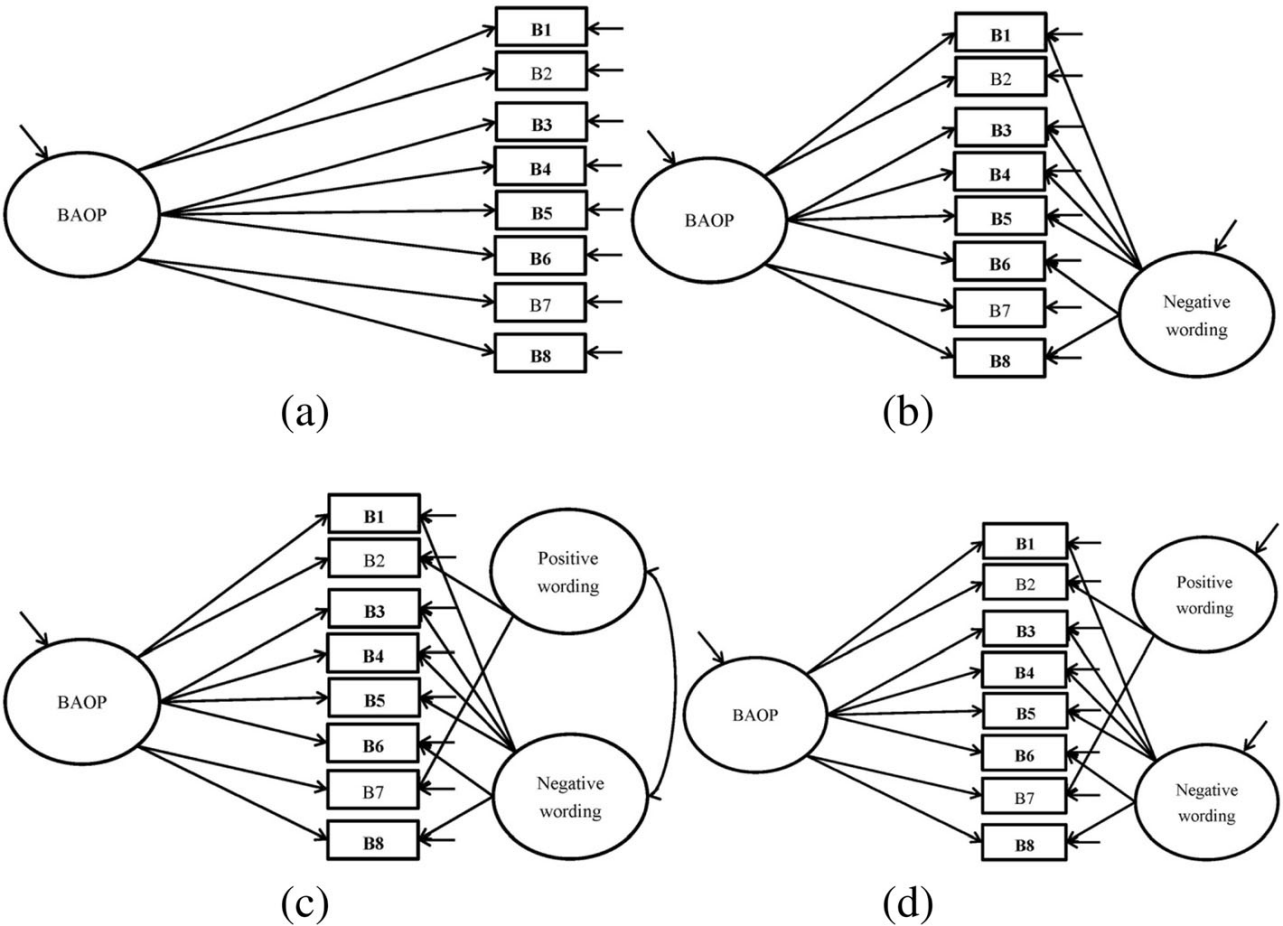
Structure of Belief About Obese Persons Scale (BAOP). **a** One-factor solution model (BAOP). **b** One-factor solution model (BAOP) with one method effect (negative wording). **c** One-factor solution model (BAOP) with two correlated method effects (negative wording and positive wording). **d** One-factor solution model (BAOP) with two uncorrelated method effects (negative wording and positive wording). Negatively worded items are in **bold**

In the BAOP, we compared four models to confirm its unidimensional structure: a one-factor structure without wording effects (Fig. 2a), a one-factor structure (BAOP) with one wording factor (negative wording; Fig. 2b), a one-factor structure (BAOP) with two correlated wording factors (positive and negative wordings; Fig. 2c), and a one-factor structure (BAOP) with two uncorrelated wording factors (positive and negative wordings; Fig. 2d). All the models were examined using the following fit indices to decide whether they are supported: comparative fit index (CFI) and Tucker-Lewis index (TLI) > 0.95 [33, 34]; root mean square error of approximation (RMSEA) < 0.06 [35]; standardized root mean square residual (SRMR) < 0.08 [36]. In addition to using the aforementioned to fit indices, we adopted expected cross-validation index (ECVI) to compare these CFA models, and a lower value of ECVI indicates a better model [26]. If the fit indices in the models with wording effect(s) (i.e., Fig. 1c to e for ATOP; Fig. 2b to d for BAOP) outperformed he models without wording effect (i.e., Fig. 1a and b for ATOP; Fig. 2a for BAOP), we might conclude that ATOP or BAOP contains wording artifacts.

After the best model among the five ATOP models and the best among the four BAOP models were confirmed, we used the two best models (one in the ATOP and another in the BAOP) to test the measurement invariance across subcultures (Hong Kong and Taiwan). According to a review [29], we constructed three submodels for the best models in the ATOP and BAOP: a configural model that did not constrain any factor loadings or item intercepts across Hong Kong and Taiwan samples; a loading constrained model that constrained all the factor loadings but not item intercepts across Hong Kong and Taiwan samples; and a loading and intercept constrained model that constrained all the factor loadings and all the item intercepts across Hong Kong and Taiwan samples. We then compared loading constrained model to configural model, and loading and intercept constrained model to loading constrained model. The measurement invariance is supported when ∆CFI > -0.01, ∆RMSEA< 0.015, and ∆SRMR< 0.01 in the model comparisons [37]. However, some argue that when the constrained model has satisfactory fit indices (i.e., CFI > 0.95, RMSEA< 0.06, and SRMR< 0.08), we still can claim the measurement invariance as supported even the values of ∆CFI, ∆RMSEA, and ∆SRMR are unsatisfactory [38]. Moreover, we used the best models to investigate the Pearson correlation between the latent ATOP and latent BAOP constructs to demonstrate the concurrent validity, and we expected the correlation above 0.3; i.e., a moderate correlation [36].

In addition, we used analysis of variance to compare the ATOP and BAOP scores among the participants who had different self-perceived weight status. Specifically, we compared whether the ATOP and BAOP scores were different among self-perceived overweight, self-perceived normal-weight, and self-perceived underweight groups. Bonferroni adjustment was applied to the post hoc comparisons.

IBM SPSS 23.0 (IBM Corp., Armonk, NY) was used to perform descriptive and inferential statistics for participants’ characteristics and the differences in ATOP and BAOP scores. R software was used to conduct CFAs and internal consistency: CFAs using the lavaan package [39] and internal consistency using Psych package [40].

## Results

There were slightly fewer male participants than female participants. More than half of the Hong Kong participants were majoring in a health-related program, whereas less than one third of the Taiwan participants were majoring in health-related program. The Hong Kong participants had significantly lower BMI values than did the Taiwan participants. No significant difference was found in age between Hong Kong and Taiwan participants (Table 1). The Hong Kong participants had lower scores than did the Taiwan participants on both the ATOP and BAOP; however, a significant difference was found in BAOP (Cohen’s *d* = 0.24) but not in the ATOP (Cohen’s *d* = 0.07).

**Table 1:** Characteristics of Hong Kong and Taiwan participants (*N* = 707)

	Hong Kong (*n* = 400)	Taiwan (*n* = 307)	*χ*^*2*^, *t, or Z* (*p*-value)
Age (year), *M* ± *SD*	20.22 ± 1.57	20.34 ± 2.04	0.15 (.88)
Gender (Male), *n* (%)	177 (44.3)	150 (48.9)	1.48 (.22)
Major (Health-related), *n* (%)	217 (54.3)	90 (29.3)	43.95 (<.001)
BMI (kg/m^2^), *M* ± *SD*	20.63 ± 3.22	21.57 ± 3.27	4.45 (<.001)
ATOP (Likert-type), *M* ± *SD*	71.76 ± 13.70	72.71 ± 13.13	0.93 (.35)
BAOP (Likert-type), *M* ± *SD*	20.16 ± 5.56	21.59 ± 6.41	3.23 (.001)

BMI Body mass index, *ATOP* Attitudes toward obese persons, *BAOP* Beliefs about obese persons

The Cronbach’s α calculated from the entire sample (i.e., Hong Kong and Taiwan participants) in the ATOP was 0.67 for Different Personality factor; 0.56 for Social Difficulties factor; 0.65 for Self-Esteem factor; 0.79 for the entire ATOP. The Cronbach’s α calculated from the entire sample in BAOP was 0.61. Table 2 further reported the Cronbach’s α calculated from the Hong Kong and Taiwan samples separately. Apart from Cronbach’s α, we calculated the McDonald’s ω for both ATOP (Entire sample: 0.86; Hong Kong sample: 0.79; Taiwan sample: 0.84) and BAOP (Entire sample: 0.78; Hong Kong sample: 0.75; Taiwan sample: 0.85).

**Table 2:** Internal consistency using Cronbach’s α for Attitudes toward obese persons (ATOP) and Beliefs about obese persons (BAOP) scales

	Entire sample	Hong Kong sample	Taiwan sample
ATOP	0.79	0.80	0.78
Different Personality	0.67	0.68	0.68
Social Difficulties	0.56	0.56	0.59
Self-Esteem	0.65	0.59	0.66
BAOP	0.61	0.57	0.65

The CFA results of the three-factor structure of the ATOP were unsatisfactory: CFI = 0.862, TLI = 0.842, RMSEA (90% CI) = 0.072 (0.067, 0.077), and SRMR = 0.078. Additionally, the one-factor structure of the ATOP was not supported (Detailed fit indices information in Table 3). However, after considering the wording effects in the one-factor structure, all the fit indices were substantially improved (CFI = 0.904 to 0.986, TLI = 0.888 to 0.982, RMSEA = 0.024 to 0.060, and SRMR = 0.041 to 0.049). Among all the models, the model with one ATOP trait and two correlated-wording-method factors performed the best. In addition, the model had the smallest value in ECVI (0.476 vs. 0.597 to 1.601). Similar findings in terms of the wording effects were shown in the BAOP. Although all the BAOP models had satisfactory fit indices (Detailed fit indices information in Table 3), the model with one BAOP trait and two correlated-wording-method factors outperformed other models, including the smallest value of ECVI (0.024 vs. 0.103 to 0.119; Table 3).

**Table 3:** Testing wording effects for Attitudes Toward Obese Persons (ATOP) and Beliefs About Obese Persons (BAOP) using confirmatory factor analysis (*N* = 707)

	ATOP				BAOP			
	M1	M2	M3	M4	M1	M2	M3	M4
*χ*^*2*^ (*df*)	1040.72 (170)*	580.25 (163)*	211.38 (149)^#^	297.82 (150)*	51.97 (20)*	46.42 (18)*	11.32 (11)*	24.33 (12)*
CFI	0.799	0.904	0.986	0.966	0.958	0.963	1.000	0.984
TLI	0.776	0.888	0.982	0.957	0.941	0.942	0.999	0.962
RMSEA (90% CI)	0.086 (0.081, 0.091)	0.060 (0.055, 0.066)	0.024 (0.016, 0.032)	0.038 (0.031, 0.044)	0.048 (0.032, 0.064)	0.047 (0.031, 0.064)	0.006 (0.000, 0.040)	0.038 (0.015, 0.060)
SRMR	0.092	0.069	0.041	0.049	0.050	0.047	0.024	0.035
ECVI	1.601	0.963	0.476	0.597	0.119	0.117	0.087	0.103

*df* degree of freedom, *CFI* Comparative fit index, *TLI* Tucker-Lewis index, *RMSEA* Root mean square error of approximation, *SRMR* Standardized root mean square residual, *ECVI* Expected cross-validation index

*p < .001; ^#^*p* = .001

M1: One trait factor (ATOP or BAOP) model

M2: One trait (ATOP or BAOP) and one method (negative wording) factors model

M3: One trait (ATOP or BAOP) and two correlated-method (positive and negative wordings) factors model

M4: One trait (ATOP or BAOP) and tow uncorrelated-method (positive and negative wordings) factors model

Because of the excellent fit indices, we additionally tested the measurement invariance across Hong Kong and Taiwan for both the ATOP and BAOP using the model with one trait (ATOP or BAOP) and two correlated-wording-method factors (Table 4). A slightly high value was found in ∆RMSEA (0.019) when we tested the invariance of factor loading in the ATOP. In addition, ∆CFI (− 0.010) and ∆SRMR (0.007 and 0.003) both supported the invariance of factor loadings and item intercepts in the ATOP. In terms of the BAOP, only invariance of factor loading was supported by ∆CFI (− 0.004) but not by other fit indices (∆RMSEA = 0.017, ∆SRMR = 0.013); invariance of item intercept was not supported by any fit indices (∆CFI = -0.029, ∆RMSEA = 0.026, and ∆SRMR = 0.012). Moreover, after controlling for wording effects, the latent construct of the ATOP was moderately associated with the latent construct of BAOP (*r* = 0.356; *p* < 0.001). Specifically, the model used for assessing correlation between ATOP and BAOP loaded all negatively worded items from ATOP and BAOP on the single negative wording construct; all positively worded items from ATOP and BAOP on the single positive wording construct.

**Table 4:** Measurement invariance across Hong Kong (*n* = 400) and Taiwan (*n* = 307)

	ATOP			BAOP		
Model fit indices	M3_1	M3_2	M3_3	M3_1	M3_2	M3_3
*χ*^*2*^ (*df*)	286.06 (298)	379.45 (335)*	436.99 (352)*	16.10 (22)	38.36 (35)	66.59 (40)
CFI	1.000	0.990	0.980	1.000	0.996	0.967
RMSEA (90% CI)	0.000 (0.000, 0.017)	0.019 (0.002, 0.029)	0.026 (0.017, 0.034)	0.000 (0.000, 0.029)	0.017 (0.000, 0.043)	0.043 (0.024, 0.061)
SRMR	0.046	0.053	0.056	0.027	0.040	0.052
Model comparison		vs. M3_1	vs. M3_2		vs. M3_1	vs. M3_2
∆χ^2^ (∆*df*)		93.38 (37)*	57.54 (17)*		22.27 (13)	28.23 (5)*
∆CFI		−0.010	−0.010		−0.004	−0.029
∆RMSEA		0.019	0.007		0.017	0.026
∆SRMR		0.007	0.003		0.013	0.012

*df* degree of freedom, *CFI* Comparative fit index, *TLI* Tucker-Lewis index, *RMSEA* Root mean square error of approximation, *SRMR* Standardized root mean square residual, *ECVI* Expected cross-validation index

*p < 0.05

M3_1: Configural model (all the factor loadings and item intercepts were relaxed across Hong Kong and Taiwan)

M3_2: Constrained factor loading model (all the factor loadings were constrained; all the item intercepts were relaxed across Hong Kong and Taiwan)

M3_3: Constrained factor loading and item intercept model (all the factor loadings and item intercepts were constrained across Hong Kong and Taiwan)

In the comparisons between the three groups in different self-perceived weight status, we found that self-perceived overweight group had significantly lower ATOP score than did the self-perceived normal-weight group (difference = 3.02; *p* = 0.04); self-perceived underweight group had significantly higher BAOP score than did the self-perceived normal-weight and self-perceived overweight groups (difference = 1.62 and 1.41, respectively; *p* < 0.05).

## Discussion

Our study examined the psychometric properties of two commonly used instruments of weight bias (ATOP and BAOP) and extended the usage for East Asian populations. Specifically, we found that both instruments had a justifiable unidimensional structure, though the wording effects should be taken into account. In addition, we found that the ATOP and BAOP were not completely measurement invariant across Hong Kong and Taiwan university students. Moreover, as our results showed that some domains in the ATOP and the entire BAOP had low internal consistencies, further refinements are needed to improve both instruments when used in Hong Kong and Taiwan populations. Despite this, the use of the entire ATOP may be appropriate because the single-factor structure of ATOP outperformed its three-factor structure.

Given the limited evidence on the psychometric properties of the ATOP and BAOP, we can only compare our results to two previous studies [17, 18]. Nevertheless, our findings somewhat corresponded to the previous evidence. Regarding the correlation between the ATOP and BAOP, Allison et al. [17] found moderate associations (*r* = 0.40 to 0.45) between the two instruments; Dedeli et al. [18] reported moderate correlations (*r* = 0.54 to 0.68); our findings on the correlation between the ATOP and BAOP latent constructs was moderate as well (*r* = 0.356). Specifically, the correlation found in our study controlled for the wording effects. The correlation between the ATOP and BAOP corresponds to the health psychology theories (e.g., Theory of Planned Behavior) that attitude is correlated with belief [41]. However, some may argue that the moderate correlation between the ATOP and BAOP is not strong enough to declare their validity. Nevertheless, our results clearly showed that the scores of ATOP and BAOP significantly differed in the groups with different self-perceived weight status. This finding corresponded to recent studies showing that self-perceived overweight adolescents might have more weight bias [42, 43]. Therefore, we suggest that using this as an external criterion, our results supported the validity of ATOP and BAOP in measuring weight bias in youth.

Our study showed that the Cronbach’s α was over 0.7 for the entire ATOP, which is an acceptable value [44]. However, the internal consistency for the entire BAOP and some domains of the ATOP was unsatisfactory. As compared to previous studies, the psychometric performance of our Chinese versions of ATOP and BAOP was somewhat inferior to those of the English (α = 0.80 to 0.84 for the ATOP and 0.65 to 0.82 for BAOP in diverse American samples [17]) and Turkish versions (α = 0.86 for the ATOP and 0.84 for BAOP [18]). However, the low internal consistencies can be justified by the two considerations: (1) Cronbach’s α increases with the number of items in a scale; therefore, the unsatisfactory results for some ATOP domains and BAOP may due to the few number of items; (2) given our study aim is to use ATOP and BAOP for heterogeneous sample (e.g., Hong Kong and Taiwan people), it is acceptable to have a Cronbach’s α value lower than 0.7.

Moreover, our CFA results may demonstrate the impact of wording effects. The CFA model that did not account for wording effects performed the worst as compared with other CFA models taking wording effects into account. Specifically, even if we reverse recoded the scores of negatively worded items to align their directions to the scores of positively worded items; the reverse recoded scores may not be comparable to the scores of positively worded items. For example, if a respondent strongly agrees with the item “Most obese people feel that they are not as good as other people” the respondent would not necessarily strongly disagree with an item worded, “Most obese people feel that they are as good as other people”. Instead, the respondent might only agree in the later item. Based on the CFA results, we suggest that the common practice of treating the ATOP and BAOP as unidimensional structures [22, 23] may be acceptable. In other words, the ATOP and BAOP scoring methods can accurately assess attitudes toward and beliefs about people with obesity.

Another important finding from the CFA is that Hong Kong and Taiwan university students may not interpret the items of ATOP and BAOP, although both were compatible with a unidimentional structure, completely in the same way, given that the measurement invariance was not supported by all the fit indices. We proposed several possibilities for the different interpretations between Hong Kong and Taiwan university students. First, the subcultures as well as Confucian philosophy, political and economic systems may have a significant role in social values, which might give participants in Hong Kong and Taiwan different attitudes toward obesity. Although both areas are affiliated with Chinese culture, Hong Kong and Taiwan have been colonized by the UK and Japan, respectively, for a long time [45]. Different colonizers’ cultures differentiated the education systems in these two areas [46] and subsequently led to dissimilar comprehension in phrases or sentences. Second, the primary languages used in Hong Kong and Taiwan are different. Although both Hong Kong and Taiwan university students were able to speak Mandarin, most Hong Kong university students communicate in Cantonese in their daily lives. In contrast, a great proportion of Taiwan students speak Mandarin and Taiwanese interchangeably in their daily lives. As a result, the use of different spoken languages was likely to influence their interpretations of the items. Future studies are thus warranted to investigate our aforementioned postulations. Third, the majors in universities were different between the participants of Hong Kong and those of Taiwan. In Hong Kong, 54.3% majored in health-related disciplines, compared to 29.3% in Taiwan. Education and knowledge about health might affect participants’ sensitivity or attitudes towards obesity and stigma, which may explain why samples in Hong Kong and Taiwan might have interpreted items on ATOP and BAOP somewhat differently.

There are some limitations in the study. First, all the participants were university students. Therefore, both Hong Kong and Taiwan samples represented populations with a high level of education. Given that educated people may have better health literacy [47], their attitudes and beliefs might not be representative of those with a low level of education. Second, the convenience sampling conducted in a small number of universities might also restrict the generalizability of our results. Third, although we observed that wording was a potential method effect in the factorial structure, we did not conduct an experimental study to confirm such an effect. Specifically, an experimental study comparing the current version of the ATOP (or BAOP) to the ATOP (or BAOP) with all items worded positively could provide useful evidence. However, such an experimental study is outside of the scope of our investigation, and we encourage future studies to use experimental designs. Fourth, as we did not collect other data that can be used as external criteria, other than self-perceived weight status, we were unable to examine the convergent and discriminant validity for both the ATOP and BAOP. Furthermore, as we did not collect the ATOP and BAOP at different time points, we were unable to investigate the test-retest reliability for both instruments. Given that convergent validity, discriminant validity, and test-retest reliability are important information for an instrument, future studies are strongly recommended to test these properties. Fifth, we acknowledged somehow the existence of cultural differences in Hong Kong and Taiwan. This may explain less than desirable measurement invariance results, which may be improved if a transadaptation method being applied to modify the items. Last, different educational backgrounds (i.e., health-related vs. non-health-related undergraduate training) in the samples of Hong Kong and Taiwan may partly account for the result of measurement variance in this study. Replication of this study is recommended, controlling for the potential confounding effects of demographic characteristics, such as educational backgrounds. Future studies will contribute to further clarifying if Chinese versions of ATOP and BAOP could be used both in Hong Kong and Taiwan.

## Conclusion

In summary, the Chinese versions of both the ATOP and BAOP might have suboptimal psychometric properties because of their low internal consistency, especially the subscales in the ATOP and the entire BAOP. However, the suboptimal internal consistency can be justified by the small number of items in ATOP domains and BAOP. Also, the use of the ATOP and BAOP across heterogeneous sample justifies the low internal consistency. Although the common usage of the ATOP and BAOP as a unidimensional structure can be supported by our findings, future refinements are necessary for strengthening both instruments. We believe that further studies on weight bias using reliable measurements are needed in Hong Kong and Taiwan to fill the literature gap.

## Data Availability

The datasets used and/or analyzed during the current study are available from the corresponding author on reasonable request.

## Abbreviations

ATOP: Attitudes Toward Obese Persons
BAOP: Beliefs About Obese Persons
CFA: Confirmatory factor analysis
CFI: Comparative fit index
ECVI: Expected cross-validation index
RMSEA: Root mean square error of approximation
SRMR: Standardized root mean square residual
TLI: Tucker-Lewis index
